# Quantitative assessment of radiation force effect at the dielectric air-liquid interface

**DOI:** 10.1038/srep20515

**Published:** 2016-02-09

**Authors:** Otávio Augusto Capeloto, Vitor Santaella Zanuto, Luis Carlos Malacarne, Mauro Luciano Baesso, Gustavo Vinicius Bassi Lukasievicz, Stephen Edward Bialkowski, Nelson Guilherme Castelli Astrath

**Affiliations:** 1 Universidade Estadual de Maringá, Departamento de Física, Maringá, PR 87020-900, Brazil; 2 Universidade Tecnológica Federal do Paraná, Departamento de Física, Medianeira, PR 85884-000, Brazil; 3 Utah State University, Department of Chemistry and Biochemistry, Logan, UT 84322-0300, USA

## Abstract

We induce nanometer-scale surface deformation by exploiting momentum conservation of the interaction between laser light and dielectric liquids. The effect of radiation force at the air-liquid interface is quantitatively assessed for fluids with different density, viscosity and surface tension. The imparted pressure on the liquids by continuous or pulsed laser light excitation is fully described by the Helmholtz electromagnetic force density.

The correct form of the momentum of light within dielectric materials and the effects caused by the radiation forces when light passes through adjacent media have been extensively debated for over a century^1^,^2^,^3^,^4^,^5^,^6^,^7^,^8^,^9^. Although the radiation pressure effects were predicted in 1871^10^, and experimentally observed in 1900^11^, a dilemma was created by controversial interpretations of the theories proposed by Minkowski in 1908^12^ and Abraham in 1909^13^ to explain the energy-momentum tensor of light. The history of the Abraham-Minkowski dilemma is intimately linked to the difficulties in sensing and interpreting the effects produced by radiation forces, which led to erroneous interpretations favoring one of the theories. This is based on the fact that both momentum descriptions have simple forms when light is incident from free space on a transparent and non-dispersive dielectric medium; Minkowski predicts a momentum in the medium proportional to its refractive index (*n*) and the photon momentum in the vacuum 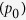 as 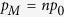, while Abraham predictions is in the form 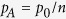. 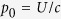, *U* is the energy of light and *c* is the speed of light. The Minkowski-Abraham controversy has theoretically been resolved by the correct division of momentum between field and medium^1^.

Early experimental investigations pursued answers to the dilemma, and continued to shed light on to this controversy. A number of reviews discuss these early experiments in details^1^,^2^,^3^,^4^,^5^,^6^,^7^,^8^,^9^, although the conclusions derived favor either theory. For instance, Jones and coauthors^14^ showed that a mirror submerged in a medium experiences a force consistent with each photon having the Minkowski momentum. Ashkin and Dziedzic^15^ demonstrated that focused laser pulses created deformations of the water-air interface; the surface of the liquid experienced a net force outward from the water as predicted by Minkowski. Although, it was later assessed that the bulging of the liquid was also influenced by radial electrostriction forces^8^,^16^. Walker and coauthors^17^ measured the torque exerted on a disk suspended on a torsion pendulum. The experiments provide evidence in favor of the Abraham form. Zhang and coauthors^18^ performed experiments based on Ashkin and Dziedzic^15^ scheme. They show the interplay between Minkowski and Abraham forces illuminating water or mineral oil. On initial inspection, experimental results may appear to be in favor of one of the formulations. However, detailed analysis demonstrates explicitly and directly the equivalence of a number of different energy momentum tensors, provided the accompanying material tensor is taken into account^1^,^6^. Yet there has been so far only limited qualitative experimental tests of our understanding of radiative transfer between electromagnetic radiation and dielectric media. Quantitative measurements of the effects of radiation forces on dielectric media have attracted large interest with the advent of optical manipulation of micro-particles in fluid media and its potential application in biological systems.

Recently, Astrath and coauthors^19^ measured surface deformation at the interface air-water generated by continuous and pulsed laser excitations using the photomechanical mirror (PM) method. The displacement caused by radiation forces was quantitatively described by the theory using the Helmholtz force density. The former experiment is a significant contribution to understanding of dynamics and momentum transfer in dielectric systems. The imparted pressure was found to have the same form as that using Minkowski momentum conservation at the interface between the dielectrics; a counterpart that could be though as propagating with the electromagnetic wave, the Abraham momentum, and that which is deposited locally in the material. The former statement would agree with running theories solving the controversial points of view regarding Abraham-Minkowski momentum formulations; this identifies the Abraham momentum as the kinetic momentum and the Minkowski momentum as the canonical momentum^1^. Here, we measure precisely nanometer scale surface deformation using the photomechanical mirror method for a systematic study to assess quantitatively the effect of radiation force at the air-liquid interface of fluids with different physical properties. Additional measurements are performed to test Zhang’s observations on the interplay between Abraham and Minkowski momenta.

## Theory

### Forces at a dielectric interface

The ponderomotive forces acting on a dielectric subjected to a non-uniform electric field can be written in terms of the stress tensor 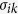 and the momentum density 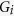 in the form^20^


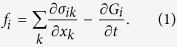


For a dielectric fluid in the absence of free charge and current, the momentum density is 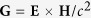 and 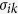 is given by


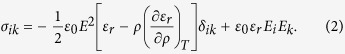


The first term in Eq. 2 accounts for electrostriction. Eq. 2 leads to a force density^8^,^13^,^20^


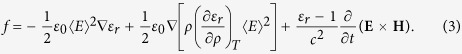


**E** and **H** describe the electric and magnetic fields, 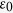 is the permittivity in vacuum, 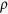 is the mass density and 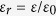 is the relative permittivity of the medium.

The first term in Eq. 3 appears in both Minkowski and Abraham energy-momentum tensor formulations. This force acts where relative permittivity presents spatial variation. The second term accounts for the deformation (electrostriction) caused by the field inhomogeneity. The last term is known as the Abraham force density. This term is supposed to average to zero at optical frequencies and can be neglected in our model. In our experiments, the Abraham and Minkowski expression for the force are identical. This fact makes the Minkowski tensor, with the inclusion of the electrostriction term, an attractive formulation for experiments in optics^6^,^21^. Thus, Eq. 3 reduces to the Helmholtz force^20^,^22^.

Here, we are considering a laser beam normally incident from air onto a flat surface of a dielectric liquid. The pressure *P* imparted by the surface force can be calculated by integrating the normal component of **f** across the interface air/liquid as


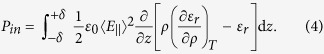



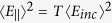
 is the electric field tangential to the surface of the liquid, 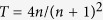 is the transmission coefficient, and 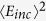 is the incident electric field. In the limit of 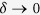, Eq. 4 results in a pressure 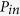 pushing the surface inwards as


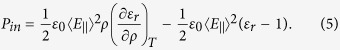


The first term in Eq. 5 is the surface contribution of the electrostriction force, and the second term is numerically as the radiation pressure defined in the Minkowski momentum transfer formulation. The radial volume electrostriction force is


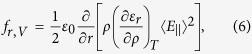


which counterbalances the inward displacement of the surface by the hydrostatic pressure 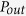
8,19,23


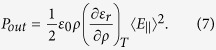


It leads to an overall pressure that elevates the surface of the liquid as^23^


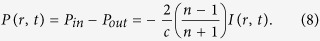


The field intensity is 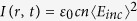. In fact, the volume contribution of the electrostriction is canceled out by its surface contribution^21^, and surface deformation is described by that due to the Minkowski-Abraham term as well as those due to gravity and surface tension^21^,^24^. 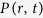 is an outward pressure effectively expanding the fluid, which is equivalent to assuming that the averaged momentum per photon is given by the Minkowski momentum^8^ as the total propagating momentum. However, the Minkowski momentum can be thought as a sum of the Abraham momentum and the mechanical momentum of the medium^6^,^21^.

### Surface deformation due to radiation forces

The pressure imparted on the liquid causes the displacement of its surface. Assuming that thermal effects caused by the laser absorption in the liquid is negligible for the overall surface deformation, the deformation can be calculated by solving the Navier-Stokes equation with appropriated boundary conditions. We used the finite element analysis (FEA) method for the numerical calculations using the software Comsol Multiphysics 4.3b with the “Laminar Two-Phase Flow, Moving Mesh” module for incompressible flow. This model solves the following equation


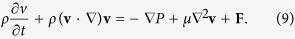


**v** describes the flow velocity, *P* is the pressure, *μ* is the dynamic viscosity, and **F** is the volume force. The pressure 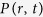 acts on the surface at 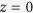 parallel to the excitation beam. A complete FEA description is presented in ref. 19. The intensity distributions of the Gaussian excitation beams, continuous-wave and pulsed, modeled here are


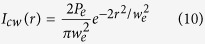


for the cw excitation, and


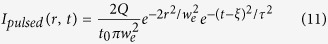


for the pulsed excitation^25^. *τ* is the pulse width, 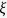 is the time to the maximum irradiance for the Gaussian pulse, 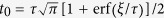 is a normalization parameter, *Q* and 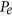 are the pulse laser energy and continuous laser power, respectively, and 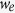 is the radius of the excitation beam in the sample. The model was built in the 2D axisymmetric geometry. The external pressure and surface tension acts on the boundary condition of the free surface. The gravity vector enters the force term as 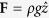 with 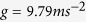 (as measured locally). Realistic sample geometry was considered (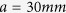 and 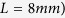. The surface displacement along the *z*-direction, 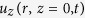, is calculated and the results are used to generate the numerical simulations for the PM signal. This process is described below.

### Photomechanical mirror

**Figure 1:**
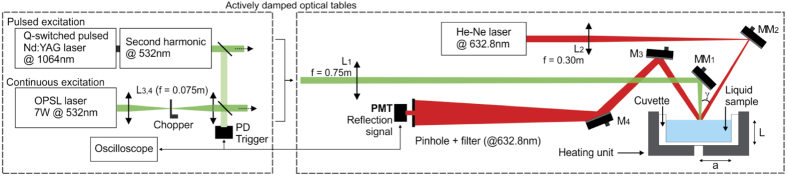
Experimental diagram for photomechanical mirror measurements. Continuous or pulsed excitation beams were focused on the sample surface. A continuous laser was arranged almost collinear to the excitation beam to probe the deformation of the liquid surface. The intensity variation of the probe beam center after reflection was detected by a pinhole-laser line filter-photomultiplier (PMT) assembly in the far-field. A digital oscilloscope triggered by the photodiode (PD) recorded the data at a repetition frequency of 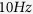 for the pulsed experiments and 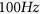 for the continuous. The apparatus was set up in separated actively damped optical tables to eliminate mechanical vibration on the liquid surface. The temperature of the samples was 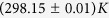. A detailed description of the experiment is presented in the Methods section.

The pump-probe PM method uses one laser to irradiate the sample normal to its surface and a low-irradiance laser to probe the sample’s surface deformation. This is performed by measuring the on-axis intensity variation of the central portion of the probe beam reflected off of the sample surface at the far-field. The symmetrical inward/outward displacement of the fluid surface converges/diverges, respectively, the probe beam at the detector, increasing/diminishing the signal at the detector. The experimental apparatuses used in this work are illustrated in Fig. 1 for continuous and pulsed excitation.

The deformation of the sample surface produces a phase shift to the reflected probe beam given by^19^


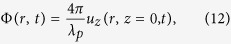


where 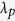 is the probe beam wavelength. Considering only the center of the probe beam spot at the detector plane in the far-field region, and using Fresnel diffraction theory, the relative intensity signal 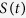 results in^19^


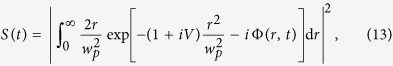


where 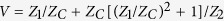, 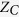 is the confocal distance of the probe beam, 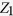 is the distance from the probe beam waist to the sample, 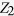 is the distance between the sample and the detector, and 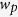 the radius of the probe beam at the sample surface. Eq. 13 can be evaluated numerically. The calculation of 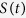 requires the determination of 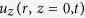 considering all the effects of the radiation forces in the liquid.

**Figure 2:**
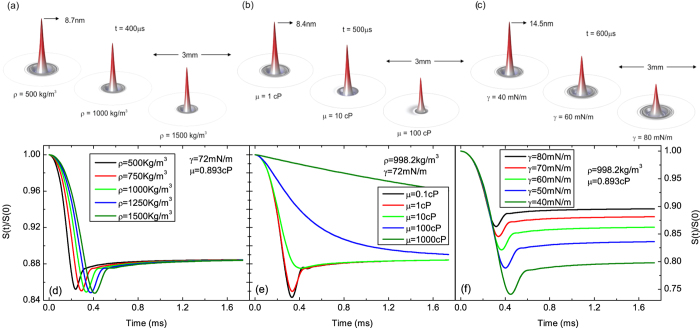
Evolution of liquid surface deformation under continuous excitation at fixed time varying (a) *ρ*–density, (b) *μ*–dynamic viscosity, and (c) *γ*–surface tension. The excitation beam radius and power were 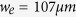 and 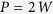, respectively, 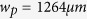, and 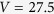. (**d**–**f**) show the corresponding PM transient signal calculated using Eq. 13, 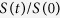.

**Figure 3:**
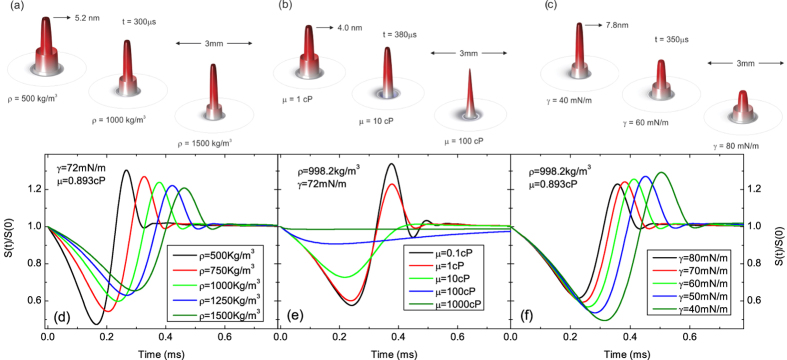
Evolution of liquid surface deformation under pulsed excitation at fixed time varying (a) *ρ*–density, (b) *μ*–dynamic viscosity, and (c) *γ*–surface tension. The excitation beam radius and energy were 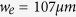 and 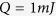, respectively, 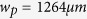, and 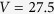. (**d**–**f**) show the corresponding PM transient signal calculated using Eq. 13, 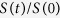.

Several calculated surface deformation and PM transient signals in Fig. 2 (cw) and 3 (pulsed) illustrate the effects of density, dynamic viscosity and surface tension on 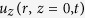 and 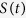. All the other parameters used in the simulations are those of water (Table 1). Figures 2 and 3(a–c) present the actual motion of the liquid[Table t2]surface at a fixed time considering different physical properties. Under continuous excitation, Fig. 2, the liquid surface rises with time reaching a maximum deformation of a few nanometers at the center of the excitation laser beam. Symmetrical waves propagate on the surface and also contribute to the convoluted intensity signal observed at the detector.

When excited by a pulse, Fig. 3, a sharp peak appears on the surface of the liquid a few *μs* after irradiation and is rapidly dispersed. The probe beam senses the entire region affected by the excitation laser, and the complex reflection pattern of the probe beam just out of the sample propagates to the detector plane. The intensity variation measured at the center of the probe beam in the far-field has a convoluted contribution from all the surface waves created on the liquid.

Figures 2 and 3(d–f) show the effect of these properties on the calculated PM transient signal. Higher density generates transients reaching the steady-state at longer times affecting slightly the amplitude signal under cw and pulsed excitations. A modification in dynamic viscosity alters the shape of the transient curves, mainly at short times. Although higher viscosity takes longer to achieve the steady-state it does not affect its final amplitude signal. Surface tension, on the other hand, affects the amplitude signal and its build-up time. The lower the surface tension, the stronger the amplitude signal.

## Results and Discussion

**Figure 4:**
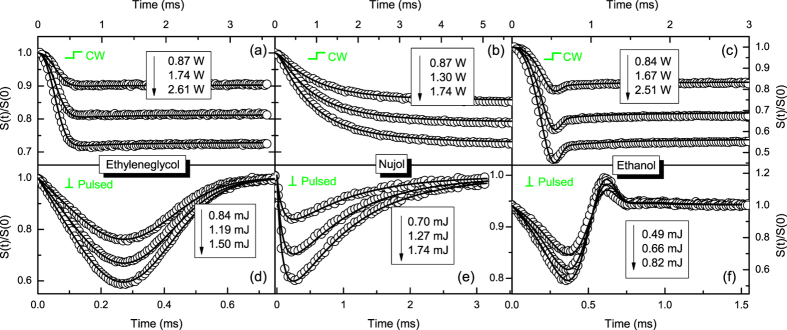
PM signal under continuous, (**a**–**c**), and pulsed, (**d**–**f**), laser excitations at 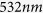 for Ethanol, Ethylene glycol, and Nujol. The transients show the intensity variation of the center of a continuous probe beam laser reflected off of the liquid surface measured at the photomultiplier tube (PMT) positioned in the far-field. Open symbols are experimental data and continuous lines represent the numerical calculations using 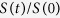 , in which 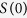 is the signal at 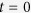. The error bars for the experimental data are smaller than 0.2%.

**Figure 5:**
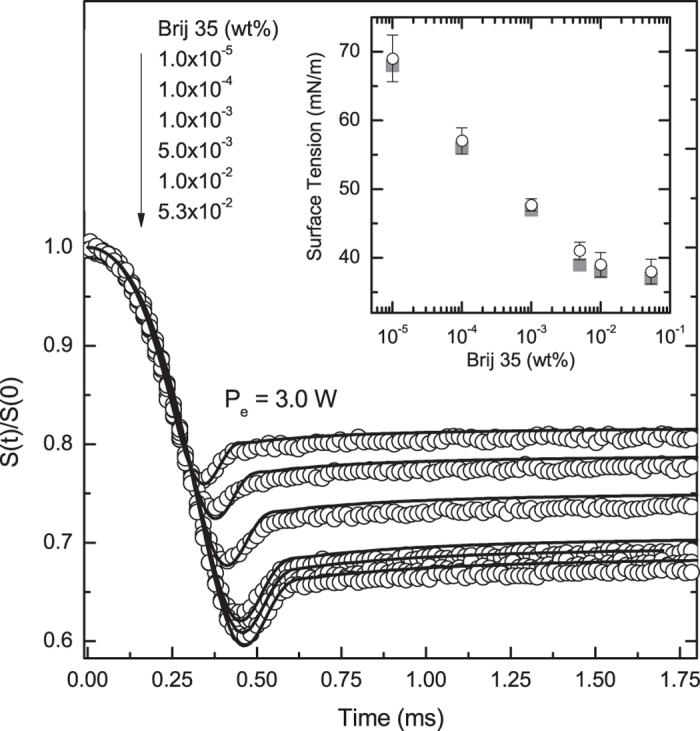
PM signal under continuous laser excitation at 532 *nm* for aqueous micellar solutions of Brij 35. Open symbols are experimental data and continuous lines represent the numerical calculations using 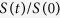. Inset shows the surface tension obtained from all numerical calculations (gray squares) and steady-state fits (open circles).

Samples with different physical properties were chosen for the experiments; Ethanol (99.9%), Nujol (99.5%), Ethylene glycol (99.5%), and aqueous solutions of 0.053 wt% to 0.00001 wt% Brij 35 [CH_3_(CH_2_)_11_(OCH_2_CH_2_)_23_OH, polyoxyethylene 23 lauryl ether]. Micellar solutions of Brij 35 were prepared by weighing the required amounts of Brij 35 in Milli-Q water. The samples were placed in a cylindrical quartz cuvette of radius 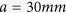 and 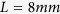 high. The sample temperature was 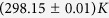. For each sample, more than 100 transients were averaged and results for the photomechanical mirror signals under continuous and pulsed laser excitations at 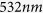 are presented in Figs 4 and 5. The transients show the intensity variation of the center of a continuous probe beam laser reflected off of the liquid surface measured at the photomultiplier tube (PMT) positioned in the far-field. Power and energy are listed in the figures. The laser beam dimensions and experimental parameters for PM setup are showed in Table 2.

Figure 4 shows PM transient signals under continuous, (a)-(c), and pulsed, (d)-(f), laser excitations for different powers and energy for Ethanol, Ethylene glycol, and Nujol. In the continuous irradiation experiments, the probe beam intensity decreases with time due to the elevation of the liquid. The surface distortion is always convex to the reflected probe beam and the corresponding signal shows a decrease in probe intensity past the pinhole at all times. As the viscosity of the samples has different order of magnitude, different shapes of transients are observed, as predicted by Fig. 2(e). For Ethanol, the probe beam intensity decreases with time for a duration of less than 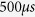 and, subsequently, a reduction in the signal towards a steady-state is observed. The same behavior are not observed for the others samples. For the pulsed excitation, the radiation force exerted in the liquid by the pulse is much shorter than the transient signal (pulse width was 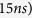. The PM sensor measures the surface wave propagating after the laser pulse. During pulsed irradiation, the surface first produces a convex column. For liquids with higher viscosity 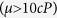 the column return for the initial condition without creating a concave surface. However, for ethanol 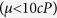, the column subsequently collapses causing a concave surface perturbation to the probe beam. This behavior corresponds to the probe laser power initially decreasing then increasing past the pinhole. The behavior observed in the experimental data also can be ascertained by the numerical simulations, as described in Fig. 3(e).

Continuous lines in Fig. 4 show the calculated PM signals. The numerical predictions are in excellent agreement for both the continuous and pulsed excitation transients. In fact, it shows quantitatively that the effects of radiation forces in liquids can be fully described by Eq. 8. The physical properties of the samples used to calculate the PM signals are listed in Table 1.

Figure 5 shows the effect of micellar solutions of Brij 35 on the surface tension of water under continuous excitation. The pattern shown on the transient curves by the addition of Brij resemble that presented in Fig. 2(f) for different surface tensions. This is, in fact, the effect that the Brij has on water; a reduction of surface tension with increasing micellar content, as presented in the inset. The continuous lines are the calculated PM signals using the parameters listed in Table 1. The only parameter susceptible to changes in the micellar solutions was the surface tension. It presented a value close to that for pure water for very low content of Brij and decreased substantially with increasing concentration of Brij.

The surface tension of the aqueous solutions can be analytically obtained from steady-state analysis. As for the air-liquid interface, the radiation pressure is compensated by the gravity and the Laplace force - the normal component of the interfacial tension applied to the curved interface^26^. It is considered that the continuous light is normally incident to the air-liquid interface at 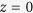 from the air to the liquid filling the half space of 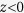. The surface displacement 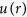 under the light radiation is given by the following equation:


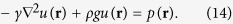


Here, 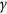 is the surface tension and the pressure 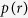 for a cw Gaussian laser beam is


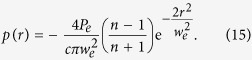


Equation 14 can be solved using Hankel integral transform as


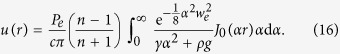



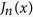
 is the Bessel Function of the first kind. The time-independent surface displacement can be used in the phase shift expression, Eq. 12, to calculate the signal, Eq. 13. From regression analysis, the surface tension of the liquid can be retrieved from steady-state signal. The results are presented in the inset of Fig. 5 and are in good agreement with the ones obtained using the time-dependent signal calculated numerically.

**Figure 6:**
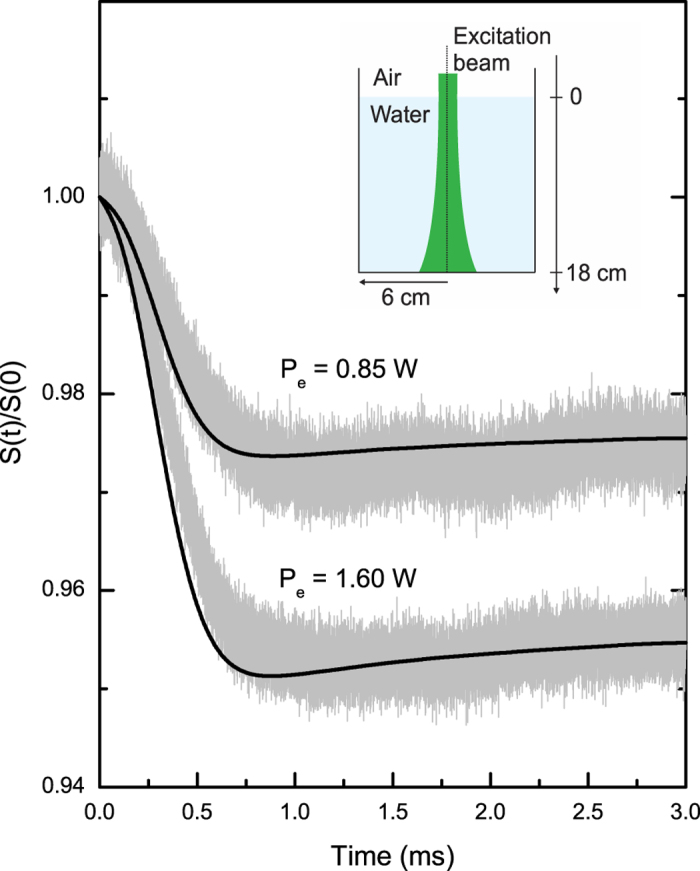
PM signal under continuous laser excitation at 532 *nm* for water. Inset shows the sample dimensions. The excitation laser beam is diverging inside the samples following the equation 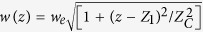 with 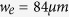, 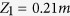, and 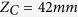. The probe beam experimental parameters were: 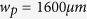, 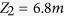, and 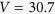.

In an attempt to replicate the findings reported by Zhang and coauthors^18^, we have performed experiments on water in air using unfocused excitation laser beam. We used a large container of water, as described in Fig. 6, and large excitation beam radius. The experimental parameters for these measurements are described in the caption of Fig. 6. These parameters reproduce the exact conditions on which the fluid would be put to motion during laser excitation and a cavitation should be seen on the water surface due to Abraham momentum transfer as predicted by Zhang and coauthors^18^. The authors state that neither the Abraham nor the Minkowski momentum is fundamental. Instead, they would emerge depending on the fluid-mechanical response of the medium to the light. With no motion, Minkowski momentum emerges; otherwise, Abraham momentum appears.

Figure 6 shows PM signals for two different excitation powers. The transients are the opposite as predicted by the authors. We can see a diverging signal that is, in fact, due to an elevation of the fluid surface. We have also performed the experiments using several different experimental parameters as well as different containers of water with different volumes. In all the tests (not shown here), the transient signal resembles the one presented in Fig. 6, i.e., an elevation of the surface of water during laser excitation. Additionally, the theoretical predictions are in very good agreement for all the experimental transients, as shown by the continuous lines in Fig. 6. For the numerical calculations, we have considered the *z*-dependence on the excitation beam radius, 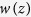, in the expressions leading to the intensity signal, Eq. 13. For this, an additional contribution to the volume electrostriction force 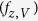 appears due to the *z*-dependence on the excitation beam radius. This contribution is written as


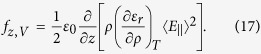


We emphasize that the PM configuration is very sensitive to very small phase shifts, which leads no doubt on the transient signals observed in our experiments. These results show that the overall effects observed are always an outward expansion of the fluid surface. For comparison, we tried to verify the findings of the authors^18^ by projecting the excitation laser beam on the wall and taking pictures at different exposure times. The beam diameter remained almost constant during laser excitation. This indicates that such a small phase shift could not be precisely measured using their experimental approach. We believe the PM method described here to be superior.

## Conclusion

In conclusion, we have experimentally demonstrated the effect of radiation forces in liquids with different physical properties. The numerical simulations are in excellent agreement with our experimental results. The expression used for the imparted pressure on the surface of the liquid from the Helmholtz force density, Eq. 3, has the same form as that using Minkowski momentum. Different experimental parameters and liquid volumes were tested and the results are all in agreement with the present theoretical description of radiation forces. Although the experiments performed here are not capable of discriminating between these two formalisms, we have assessed that for all these different materials and experimental configurations, Minkowski momentum transfer still holds and describes the surface deformation observed. However, Minkowski formulation could be as well regarded as the total momentum in the system; a sum of the momentum which propagates with the electromagnetic wave, the Abraham momentum, and that which is deposited locally in the material.

## Methods

### Photomechanical Mirror

Experimental diagram for photomechanical mirror measurements. Continuous or pulsed laser excitations are provided by TEM_00_ beams with an optically pumped semiconductor laser (Coherent, Verdi G7, 532 *nm*) or a Q-switched pulsed Nd:YAG with second harmonic TEM_00_ laser operating at 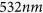 (Quantel, Brilliant B, pulse width of 15 *ns*), respectively. The excitation beams were focused on the sample surface using a 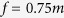 focal length lens (L_1_). A 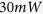 continuous TEM_00_ He-Ne laser at 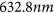 (Melles Griot, Model 25-LHR-151-249), almost collinear to the excitation beam 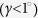, focused by lens L_2_ 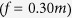, was used to probe the deformation of the sample surface. The intensity variation of the probe beam center after reflection was detected by a pinhole-laser line filter-photomultiplier (PMT) assembly in a far field (approximately 6.8 *m* from the sample surface). The laser line filter is used to prevent the excitation laser beam and ambient light from being detected by the photomultiplier tube (Hamamatsu, Model R928). The PMT was biased with a high voltage power supply (Newport, Model 70706). A digital oscilloscope (Tektronix, Model DPO4102B) recorded the data. Partial reflections from the excitation beams were used to trigger the oscilloscope by the photodiode PD (Newport, Model 818-BB-22) at a repetition frequency of 10 *Hz* for the pulsed experiments and 100 *Hz* for the continuous. A mechanical chopper (Thorlabs, Model MC2000) was used to modulate the continuous excitation. To eliminate mechanical vibration on the liquid surface, the excitation lasers, chopper and the motorized (Thorlabs, Model ZST213) alignment mirrors (MM_1_ and MM_2_) were placed in separated actively damped optical tables, as shown in the details (dashed lines). A heating unit and a temperature controller (Lakeshore, Model 340) were used to set the samples temperature to 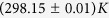. The excitation and probe beam radii were measured with a beam profiler (Thorlabs, Model BP104-UV) and a beam profile camera (Coherent, Model Lasercam HR). Laser energy and power were measured using a pyroelectric energy sensor (Thorlabs, Model ES120C) and a power meter (Spectra-Physics, Model 407A), respectively.

## Additional Information

**How to cite this article**: Capeloto, O. A. *et al*. Quantitative assessment of radiation force effect at the dielectric air-liquid interface. *Sci. Rep*. **6**, 20515; doi: 10.1038/srep20515 (2016).

## Figures and Tables

**Table 1:** Physical properties of the liquids used in the simulations.

Sample	Mass density *kgm*^−3^	Dynamic viscosity *cP*	Surface tension *mNm*^−1^	Refractive index
Ethanol	803.4	1.081	21.9	1.36
Ethylene glycol	1113.0	16.75	48.0	1.45
Nujol	864.5	109.80	32.0	1.46
Water	998.2	0.893	72.0	1.33
0.053 wt% Brij	998.2	1.04	38 ± 2	1.33
0.01 wt% Brij	998.2	1.04	39 ± 2	1.33
0.005 wt% Brij	998.2	1.04	41 ± 1	1.33
0.001 wt% Brij	998.2	1.04	47.7 ± 0.9	1.33
0.0001 wt% Brij	998.2	1.04	57 ± 2	1.33
0.00001 wt% Brij	998.2	1.04	69 ± 3	1.33

**Table 2 t2:** Experimental parameters for PM setup.

Parameters		Continuous excitation	Pulsed excitation
*Z*_1_	*mm*	290	290
*Z*_2_	*m*	6.8	6.8
*Z*_*C*_	*mm*	11.0	11.0
*V*		27.5	27.5
*ξ*	*ns*		30
*τ*	*ns*		15
*w*_*p*_	*μm*	1290	1317
*w*_*e*_	*μm*	107	133
